# Structure of the d-Cycloserine-Resistant Variant D322N of Alanine Racemase from *Mycobacterium tuberculosis*

**DOI:** 10.1021/acsbiomedchemau.2c00074

**Published:** 2023-03-27

**Authors:** Cesira de Chiara, Gareth A. Prosser, Roksana Ogrodowicz, Luiz P. S. de Carvalho

**Affiliations:** † Mycobacterial Metabolism and Antibiotic Research Laboratory, The Francis Crick Institute, London, NW1 1AT, United Kingdom; ‡ Structural Biology Science Technology Platform, The Francis Crick Institute, London, NW1 1AT, United Kingdom; § Department of Chemistry, The Herbert Wertheim UF Scripps Institute for Biomedical Innovation & Technology, Jupiter, Florida 33458, United States

**Keywords:** Alanine racemase, d-cycloserine, tuberculosis therapy, antibiotic resistance, X-ray crystallography

## Abstract

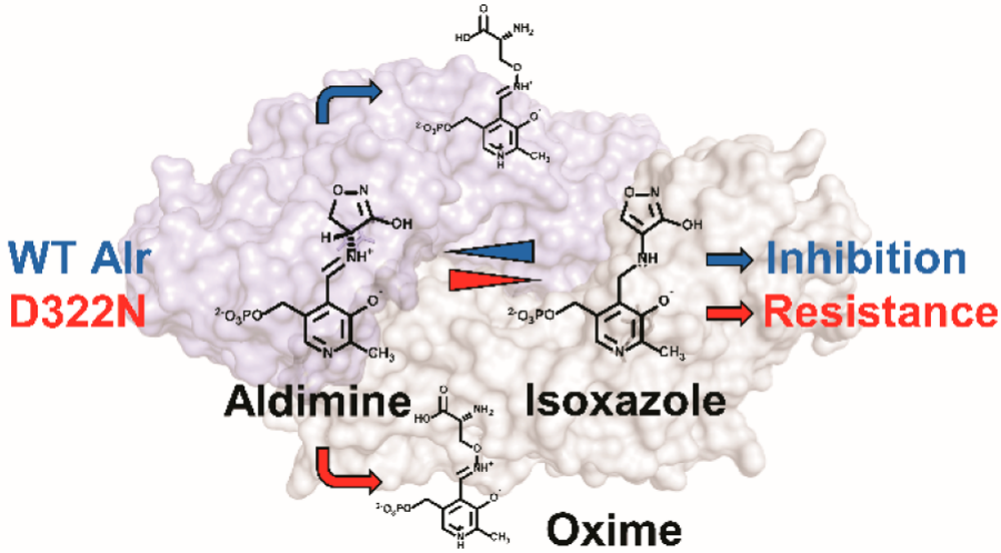

Alanine racemase (Alr) is a pyridoxal 5′-phosphate-dependent enzyme that catalyzes the racemization of l-alanine to d-alanine. Alr is one of the two targets of the broad-spectrum antibiotic d-cycloserine (DCS), a structural analogue of d-alanine. Despite being an essential component of regimens used to treat multi- and extensively drug-resistant tuberculosis for almost seven decades, resistance to DCS has not been observed in patients. We previously demonstrated that DCS evades resistance due to an ultralow rate of emergence of mutations. Yet, we identified a single polymorphism (converting Asp322 to Asn) in the *alr* gene, which arose in 8 out of 11 independent variants identified and that confers resistance. Here, we present the crystal structure of the Alr variant D322N in both the free and DCS-inactivated forms and the characterization of its DCS inactivation mechanism by UV–visible and fluorescence spectroscopy. Comparison of these results with those obtained with wild-type Alr reveals the structural basis of the 240-fold reduced inhibition observed in Alr D322N.

The antibiotic d-cycloserine (DCS) is produced by *Streptomyces lavendulae* and *S. garyphalus*.^1^ A structural analogue of d-Ala, DCS (**1**; Scheme 1) targets two essential enzymes of the bacterial peptidoglycan biosynthesis pathway, alanine racemase (Alr) and d-Ala:d-Ala-ligase (Ddl).^2^ Surprisingly, reports of resistance to DCS in strains infecting humans are scarce, despite its use in therapy for over 60 years. DCS has been described as a “cornerstone” for the treatment of multidrug (MDR) and extensively drug (XDR) resistant tuberculosis (TB)^3^ and is currently recommended for inclusion in the treatment of MDR/rifampicin-resistant (RR-)TB patients on longer regimens.^4^ Together, MDR and XDR TB cases account for half a million cases and 180,000 deaths each year worldwide and currently represent a global emergency in the therapy of tuberculosis.

**Scheme 1:**
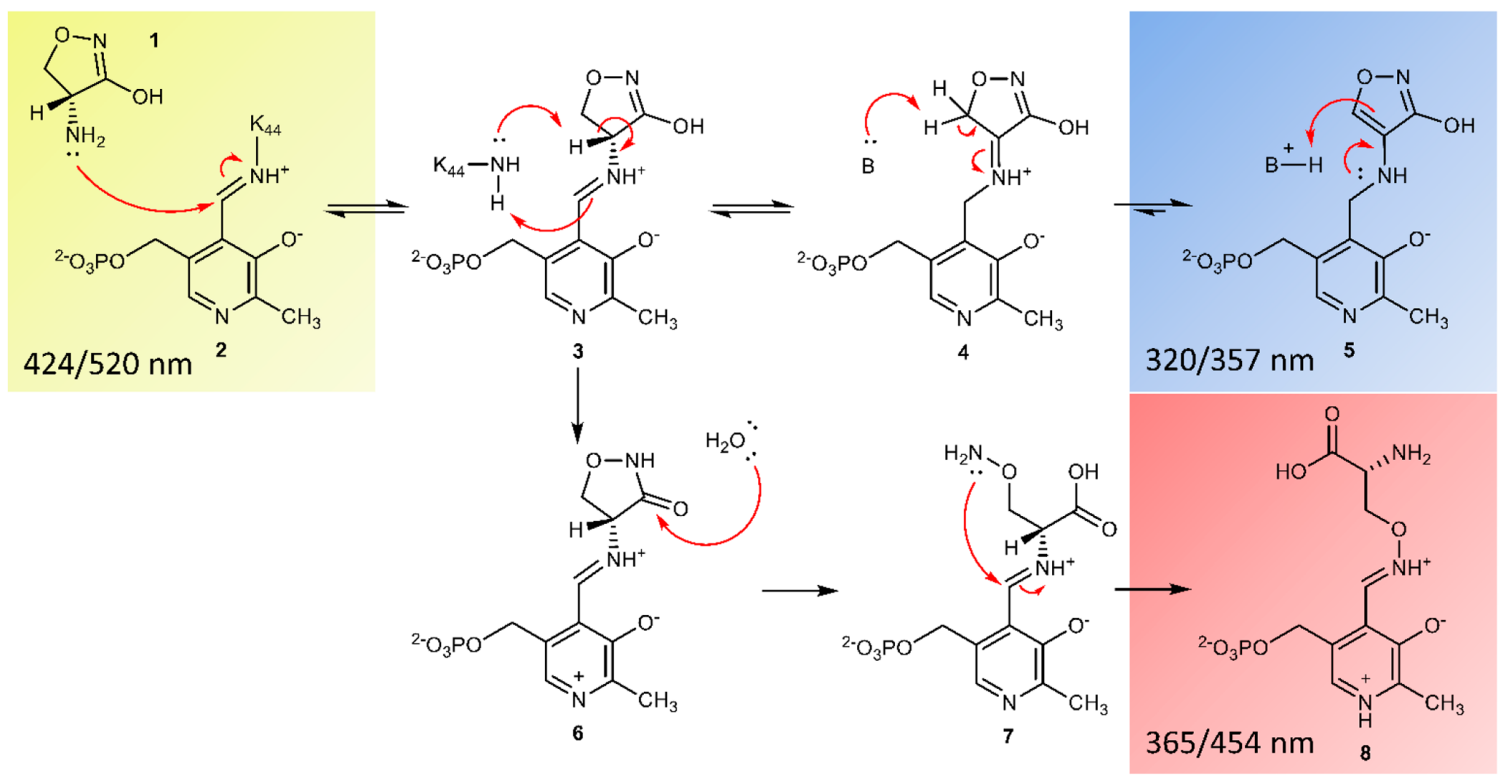
Revised Mechanism of Inhibition of Alr by DCS; Based on de Chiara et al., 2020^9^^,^ There are two pathways for the inactivation of alanine racemase (Alr) by DCS. The “isoxazole-forming pathway” from **3** to **5** happens “on-enzyme”, and all steps have been proved to be reversible. It runs alongside the irreversible “oxime-forming pathway”, from **3** to **8**. While it is possible that the conversion from **3** to **7** may happen “on-enzyme”, it is expected that **7** is released from the enzyme into solution, where it is rapidly converted to oxime **8**. The fluorescence excitation/emission wavelengths are reported for species **2**, **5** and **8**.

Recently, we investigated the biological rationale behind the lack of *M. tuberculosis* resistance to DCS and identified the ultralow rate of emergence of DCS resistance conferring mutations as the dominant biological factor, as high fitness cost mutations were also observed.^5^ We demonstrated that Alr plays a central role in DCS resistance in *M. tuberculosis*, and identified that mutation of Asp 322 to Asn (D322N) confers resistance to DCS. The D322N mutation was observed in eight out of 11 independently obtained variant strains. Kinetic characterization of the D322N variant showed that this alteration reduces the affinity (*K*_i_) of Alr for DCS 240-fold.^5^

The first structure of a DCS-inhibited Alr, that from *Geobacillus stearothermophilus*, was published almost 20 years ago.^6^ However, it was not until recently that the mechanism of inactivation, previously inferred from other PLP-dependent enzymes^7^,^8^ was investigated in detail, and specifically, in MtAlr.^9^ After demonstrating that Alr is not fully inhibited by DCS in *M. tuberculosis* treated with DCS,^10^ we determined the structure of DCS-inactivated MtAlr at high resolution and revealed the inactivation mechanism using a combination of spectroscopy, kinetics, and accurate mass–mass spectrometry.^9^ Alongside the reversible inactivation reaction, leading to the synthesis of the isoxazole-PLP adduct (**5**; Scheme 1), we identified a slower, previously unrecognized, pathway involving DCS-ring opening and partial reactivation of MtAlr (**6**–**8**; Scheme 1). *M. tuberculosis’* slow growth allows it to benefit from this intrinsic Alr reactivation mechanism, which is likely unimportant for fast-growing bacteria such as *E. coli*.

Here we present high-resolution structures of MtAlr D322N in both its active and DCS-inhibited forms, and characterize the mechanism of inhibition by UV–visible and fluorescence spectroscopy. Comparing the active site of the D322N variant to that of the wild-type enzyme reveals why this variant exhibits reduced affinity for DCS and thus resists inactivation.

We incubated MtAlr D322N with a saturating concentration (100-fold molar excess) of DCS, at 37 °C and followed the inactivation reaction by UV–visible and fluorescence spectroscopy (Figure 1). In the absence of DCS, there is an absorbance band at 424 nm due to pyridoxal 5′-phosphate (PLP) linked to K44 via an aldimine bond (internal aldimine, **2**) (Figure 1A, top). Treatment of wild-type MtAlr with DCS results in the disappearance of this band. When MtAlr D322N is treated with DCS, however, the disappearance of the band at 424 nm is incomplete and a residual low intensity signal is observed at 430 nm (Figure 1B, top). Moreover, in wild-type MtAlr, disappearance of the absorbance band due to the internal aldimine is coupled with the appearance of a band at 320 nm, due to the pyridoxamine (PMP)-like form of the cofactor, in the isoxazole derivative **5** (Figure 1A, top). In the case of MtAlr D322N, the signal at 320 nm is barely visible as a shoulder of the more intense signal of the aromatics, expected around 280 nm (Figure 1B, top). Unsurprisingly, given its lower affinity for DCS, complete inactivation determined using the changes in the fluorescence spectrum at 520 nm, is approximately ∼30-times slower with MtAlr D322N than with the wild-type enzyme (Figure S1).^9^

**Figure 1:**
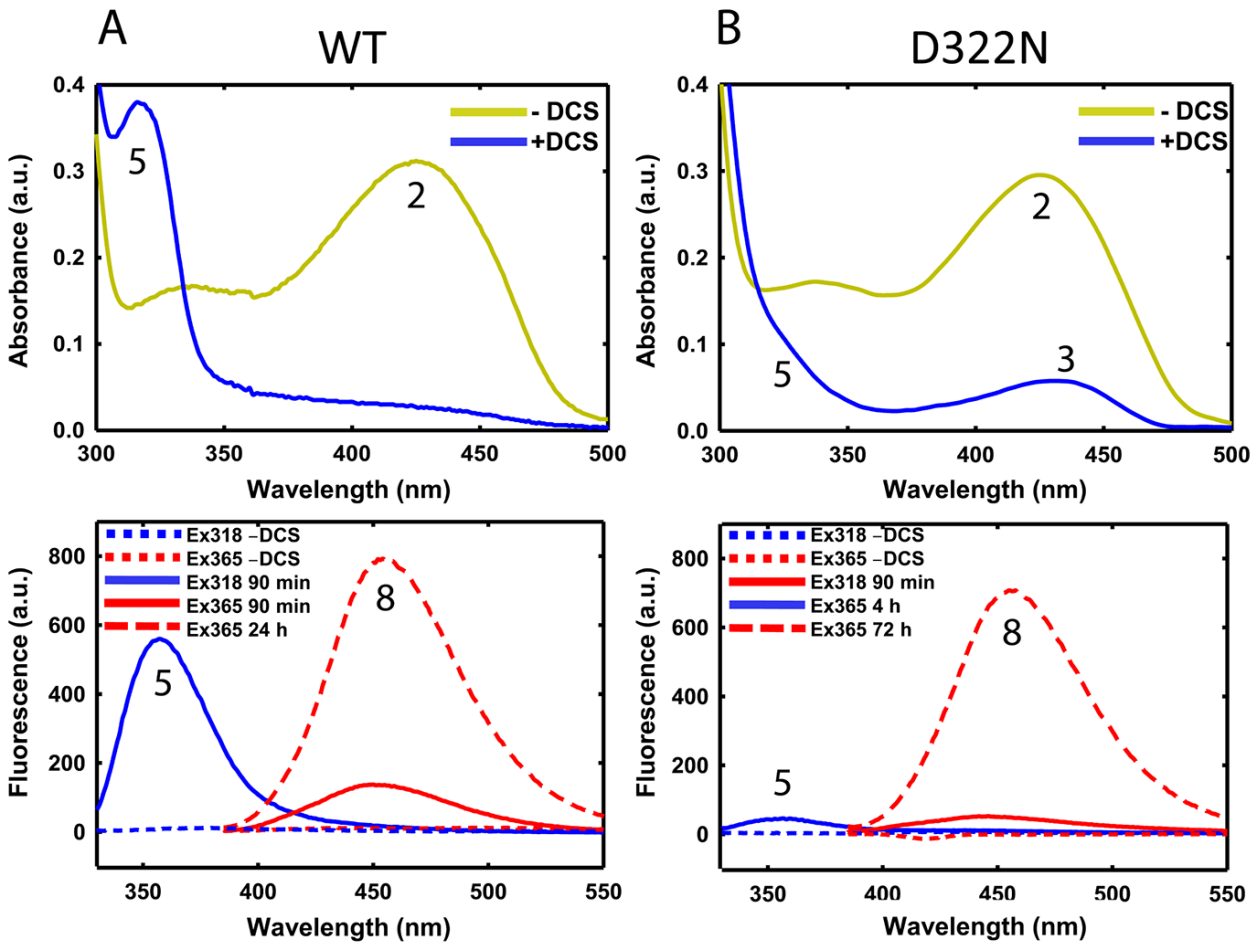
Spectroscopic analysis of wild-type and D322N Alr. UV (top) and fluorescence (bottom) spectra of active and DCS-inactivated A) wild-type MtAlr and B) variant D322N. All spectra in the presence of DCS were acquired at reaction completion, except for fluorescence spectra at 90 min of incubation (red continuous line).

When the reaction was monitored by fluorescence, the 520 nm emission band of the internal aldimine (Figure S1) decreased over time, on a comparable time scale to the disappearance of the band at 424 nm in the visible spectrum (data not shown). However, the emission of the isoxazole (**5**) at 357 nm (upon excitation at 318 nm) is significantly less intense for the variant than that observed for the wild-type MtAlr,^9^ in agreement with what we observed for the 320 nm band in the UV spectrum (Figure 1A and B). In contrast, the fluorescence band with 365 nm excitation and 454 nm emission wavelengths, typical of the substituted oxime **8** formed in the secondary pathway,^9^ is clearly visible. The rate of formation of this band is ∼3-fold lower, and it takes longer to reach completion with D322N (72 h) than with the wild-type (24 h). These findings indicate that the mechanisms of inhibition and reactivation are qualitatively conserved between MtAlr and MtAlr D322N, but exhibit some kinetic differences (Figure 1A, Scheme 1). Overall, these data suggest that, in MtAlr D322N, significantly less of the initially formed species **3** is converted into **5** than in the wild-type enzyme.

We grew crystals for uninhibited and DCS-inactivated MtAlr D322N. The latter were produced by pre-incubating MtAlr D322N with a 100-fold molar excess of DCS, for 24 h at 30 °C. As expected, the crystals of the uninhibited form showed the characteristic intense yellow color due to the emission of the internal aldimine **2** form of PLP, whereas the crystals for the DCS-inactivated enzyme appeared almost colorless (not shown). Both types of crystals, although morphologically different, shared the crystallographic space group *P*4_1_2_1_2, and diffracted to high resolution: 1.58 and 1.78 Å for the uninhibited and inhibited variants, respectively. The structures were determined by molecular replacement using the coordinates of uninhibited (PDB ID: 1XFC) and inactivated MtAlr (PDB ID: 6SCZ)^9^, respectively (Table S1). Alr enzymes are functional homodimers. Residues from both chains contribute to the active site, including the two catalytic general acid–bases K44 and Y273′ (numbering refers to MtAlr, UniProtKB entry P9WQA9) on opposite sides of PLP (Figure 2).^6,11^ In the native, or active, state of the enzyme, PLP is covalently linked to K44 via an aldimine or “Schiff’s base” type of bond (**2**). All the hydrogen bond interactions with the phosphate group and the pyridine nitrogen of PLP are formed by residues belonging to the same chain as K44, as are the additional hydrophobic interactions with residues making contacts with the pyridine ring and its substituent methyl group.^11^ The interactions with the phenolic oxygen of PLP, meanwhile, include water-mediated hydrogen bonds to fully conserved residues belonging to the adjacent protomer (indicated by a prime symbol “ ′ ”), one of which is D322′. In the structure of uninhibited MtAlr (PDB ID: 1XFC), D322′ interacts with this oxygen via one water molecule (w1) (Figure 2A).

**Figure 2:**
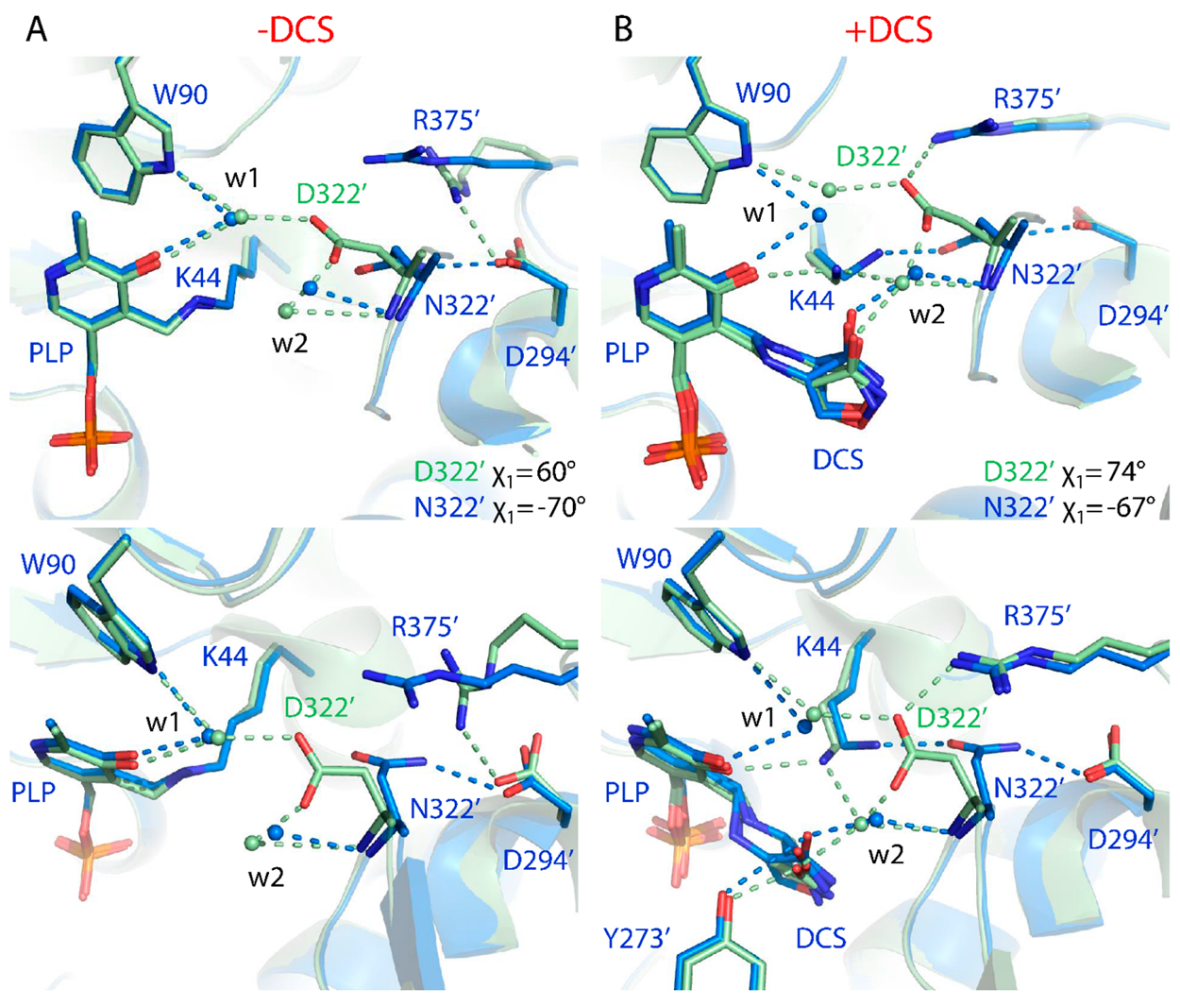
Superposition of active site “A” (catalytic K44 of chain A) of A) uninhibited and B) DCS-inactivated wild-type MtAlr and variant D322N. A conserved water (w1) is shown which in both uninhibited and DCS-inhibited wild-type MtAlr is involved in a H-bond bridging PLP “O” to D322′ “O”. A second conserved water (w2) plays a role in the DCS-inactivated structure connecting DCS “O” to D322′ via two strong H-bonds. The strongest of these two bonds between D322′ “O” and w2 is lost in variant D322N due to the different orientation of the N322′ side chain compared to D322′. In the bottom panels, a rotation by ∼70° has been applied for a better view of R375′, D322′, and N322′ side chains. The entire loop including the second catalytic residue Y273′ is disordered in site “A” of the – DCS structure of both wild-type (1XFC) and D322N variant (8AHW) and could not be located in the density (A). In contrast, the loop could be fitted into the density of the DCS-inhibited form of both wild-type (6SCZ) and the variant (8B8H) (B). The sequence numbering of uninhibited wild-type MtAlr (1XFC) has been increased by 2 to bring it into agreement with 6SCZ and the structures from the current study. This increase is consistent with that resulting from the correction of an initiation error in the UniProtKB entry P9WQA9.

Comparing wild-type MtAlr to the D322′N variant, we observe a clear conformational change in the orientation of the N322′ side-chain with respect to the wild-type D322′ (Figure 2A). In particular, the χ_1_ angle changes from a positive value of +60.3° for D322′ to a negative value of −69.7° for N322′, as measured in chain B. Interestingly, a χ_1_ value of ∼ +61.0° is statistically observed in only 10% of the aspartate side-chains in crystal structures compared to a 51% probability of a −71.0° χ_1_ value. This less energetically favored conformation is likely to be induced by the electrostatic repulsion that a D322′ with a χ_1_ ∼ −71.0° would experience with the nearby fully conserved aspartate D294′, due to the short distance of about 3 Å between them and the absence of neutralizing interactions. A salt bridge is present in both uninhibited and inhibited MtAlr between R375′ and D294′, and between R375′and D322′, respectively, but they appear unable to stabilize a negative rotamer for D322′. The abolition of such repulsion in MtAlr D322N allows N322′ to acquire a more energetically favorable negative χ_1_ value. Therefore, rather than being oriented toward PLP and W90, like the wild-type D322′, the N322′ side chain is oriented toward D294′, with N322′ making a H-bond to D294′, at 2.99 Å. As a consequence, although the water molecule (w1) originally bridging D322′ and PLP phenolic oxygen is structurally conserved in the variant and still makes a hydrogen bond to PLP, due to the longer distance the H-bond to N322′ is no longer present (Figure 2A). The interaction between PLP oxygen and Q323′, mediated by two water molecules, is maintained in MtAlr D322N (not shown).

Overall, the same conformational changes observed in the structure of wild-type DCS-inhibited MtAlr (6SCZ) are found in the DCS-inhibited MtAlr D322N structure. In particular, DCS, rather than the catalytic K44, is engaged in a covalent bond to PLP (Figure 2B).^9^ However, while isoxazole **5** is predominant in the wild-type enzyme, with only 4% of external aldimine **3** in site ‘A’, the ratio is reversed in the MtAlr D322N structure with the external aldimine becoming the dominant ligand in ‘A’ and the aldimine 3 as a sole species in ‘B’ (Figure S2). In the inhibited form, the N322′ side-chain retains the orientation observed in uninhibited MtAlr D322N, with a negative χ_1_ value of −66.2° and −66.7° for chain A and B, respectively. This torsion angle is important, as the wild-type D322′ side-chain is able to establish a water (w2)-mediated H-bond with the carbonyl group of DCS in the aldimine adduct **3** and/or the hydroxyl moiety of DCS in the hydroxyisoxazole **5** (Figure 2A). Notably, the hydrogen bond between D322′ and the conserved bridging water (w2) with a donor–acceptor distance of ∼2.0/2.2 Å in site A/B, is strong and considered to be mostly covalent in nature, and the bond between the water molecule and the isoxazole **5** is also strong (2.5/2.6 Å in A/B). Given the very short distance between w2 and D322′ in MtAlr, it is possible that either w2 or D322′ could be protonated and the proton shared between the two oxygen atoms, neutralizing the negative charge on both D322′ and isoxazole **5** in the inhibited enzyme. A similar effect can be predicted for the neutralization of the charge on the carboxylate of the substrate alanine. The structure of GsAlr (PDB ID: 1L6F) bound to a molecule that mimics a PLP-alanine-adduct clearly shows that w2 provides a link between the oxygen of Ala and D313′.^12^ The presence of a protonated water in a hydrogen bond pattern consistent with a short distance between the two water molecules has been previously identified in the active site of *Bacillus anthracis* Alr (PDB ID 2VD8, 1.47 Å), where, together with nearby R136, it neutralizes the negative charge on the phenolic oxygen and on D318′.^13^

We previously suggested that this conserved water w2, at 3.3 Å from the DCS carbonyl of the external aldimine **3**, could play a role in the hydrolysis of the DCS ring that leads to the formation of the oxime **8**, and that the short H-bond may render the carbonyl more electrophilic.^9^ Due to the orientation of N322′, no H-bond is possible between this residue and w2 due to the longer distance (4.4 Å), and the H-bond between w2 and the carbonyl is also longer (3.1 Å) in D322N than in wild-type (2.6 Å), particularly in site B. This is likely the major determinant of the lower affinity for DCS previously observed.^5^ In our previous paper, we also hypothesized that the catalytic Y273′ may contribute to the deprotonation of a second water molecule, at 4.3 Å to the carbonyl, and activate it for nucleophilic attack. We observe that in the inhibited form of the variant, Y273′ has a significantly higher B-factor than the surrounding residues, particularly in site A, as compared to the wild-type and could therefore be less efficient in this role. If Y273′ is the general base involved in the conversion of **4** to **5** (Scheme 1), this could explain not only the 3-fold slower formation of the oxime, but also the lower propensity to form the isoxazole **5**.

Of note, the N322′ oxygen is at an optimal distance to make a ∼ 2.7 Å H-bond in both sites with the K44 side chain, which then turns toward N322′ and away from the PLP-isoxazole adduct **5** compared to wild-type. This interaction contributes to additional stabilization of the catalytic base in the “resting” position, as observed in the other inhibited Alrs (for a list see Table S2), while N322′ still retains the H-bond to D294′ (Figure 2B). This could lead to the observed prevalence of **3** over **5**, as K44 is implicated in the mechanism of conversion of **3** to **5** in wild-type Alr (Scheme 1).

Lastly, we compared the structures of uninhibited and DCS-inhibited MtAlr to the structures of bacterial Alr and DadX (catabolic Alr enzymes) enzymes available in the PDB: 17 in the active form and the 7 in the DCS-inactivated form (Table S2). We noticed that the structures of MtAlr and *S. lavendulae* Alr (SlAlr, 1VFH), are the only two showing a positive value for D322′ χ_1_. This feature is associated with the nearby conserved arginine R375′ forming a salt bridge only with D294′ (in MtAlr numbering) and showing a relatively higher B-factor on average than the surrounding residues. The *G. stearothermophilus* (GsAlr) structure is shown as a representative example of all other Alr structures with negative χ_1_ in Figure S3A(14) and where the arginine forms a stable bidentate salt-bridge with the side chain of both the conserved aspartate residues, i.e. D313′ and D285′ of GsAlr (Figure 3, close-up). While considering only the structures of uninhibited MtAlr and SlAlr might suggest that positive χ_1_ values are caused by disruption of the R375′-D322′ salt bridge, we ruled out this possibility. In fact, the inhibited MtAlr that also has a positive χ_1_ shows an ordered R375′ side-chain and retains the R375′-D322′ salt bridge, while the R375′-D294′ salt bridge is disrupted. Interestingly, the positive χ_1_ angle of uninhibited SlAlr becomes negative in the inhibited form and a simultaneous salt bridge of the Arg with both the Asp side chains is observed. SlAlr, from one of the Streptomyces strains producing DCS, was previously found to be substantially less inhibited than EcAlr, indicating resistance to its own product.^15^ A summary of the observed salt bridges between arginine and aspartates is provided for all the representative structures in Table S3 and is illustrated in Figure 3 (close-up).

**Figure 3:**
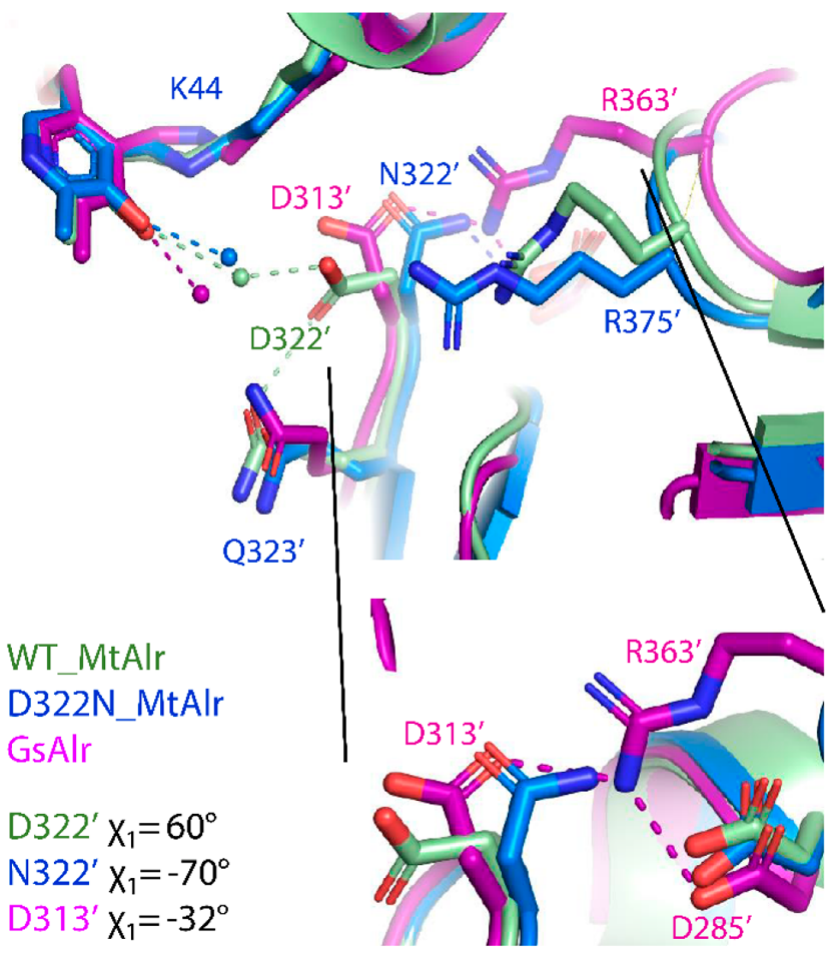
*G. stearothermophilus* Alr (GsAlr) crystal structure (PDB 1SFT) superposed on wild-type MtAlr (1XFC) and the D322N variant (this study, 8AHW). As in all the available structures of alanine racemase, with the exception of MtAlr and SlArl, D313′ of GsAlr (1SFT) retains a negative χ1 value of −32° stabilized by H-bonds of both D313′ and D285′ (D322′ and D294′ in MtAlr) with the conserved R363′ (see **close-up** view where the wild-type and variant MtAlr arginine side chains have been omitted for clarity).

It should be noted that while in MtAlr the link, mediated by w2, between the D322′ “O” and the hydroxyisoxazole **5** is disrupted by the mutation, in all the other inhibited Alrs the H-bond to w2 is preserved despite the change in the Asp rotamer. However, the distance is generally much longer in the orthologs (2.7 to 3.4 Å) than in MtAlr (∼2.1 Å). In our structural analysis of the orthologs, we have observed an interplay between this w2-mediated link to Asp, the catalytic tyrosine phenolic “O” and a conserved arginine (Y273 and R142, respectively, in MtAlr), which contribute to a variable extent to the recognition and stabilization of the alanine carboxylate or hydroxyisoxazole **5** “O”. These three contributions are finely tuned in a species-specific way, and it is generally observed that the longer the distance between the DCS “O” and the Arg, the shorter the distance between the DCS “O” and the tyrosine OH.

Although it would be interesting to evaluate whether the negative orientation of the rotamer of the other orthologs correlates with lower susceptibility to DCS and slower time-dependence of inhibition, there are no comparable data available in the literature. The only exception is for MtAlr, *Staphylococcus aureus* Alr, and *Pseudomonas aeruginosa* Alr. MtAlr has ∼9- and 1.6-fold higher affinity for DCS than SaAlr (the structure of which is not available) and PaAlr (PDB ID: 6A2F), respectively.^16^ Like all the other Alr structures, PaAlr shows a negative χ1. Although it is just one example, the comparison of MtAlr and PaAlr suggests that a negative value does correlate with lower affinity for DCS.

Similarly, there are no systematic studies on the rate of reactivation, via formation of oxime **8**, among species *in vitro* or *in vivo*. Other important factors besides the rotamer of the Asp may affect the reactivation kinetics, including the dissociation of the dimer into monomers (necessary for the release of the hydrolyzed oxime **8**) and, particularly *in vivo*, the growth rate of the specific microorganism. In fast-growing species, the growth rate can compete with the reactivation rate;^9^ consequently, the reactivation pathway is only significant for slow growing-organisms, such as *M. tuberculosis*.

In summary, we have presented a structural and spectroscopic characterization of the inhibition of the DCS-resistant MtAlr D322N. Like the wild-type enzyme, D322N shows a side reaction, which ultimately leads to the formation of the substituted oxime **8**. The crystal structures determined at high-resolution identify a change in the orientation of N322′, as compared to D322′, and the loss of a strong H-bond, as the likely cause of the observed lower affinity of MtAlr D322N for DCS. Interestingly, the original χ_1_ angle of D322′ appears to be a rare occurrence among the alanine racemase structures, and to depend on the ability of a “close in space but distant in sequence” arginine to form stabilizing salt bridges/H-bonds (Figure S3B). It is not known whether the same mutation (D322N) causes resistance to DCS in other Alr enzymes but, if this is the case, on the basis of the structural insights that we have provided in this study, we could predict that the mutation would impact affinity for DCS or processing of other Alrs significantly less than the Mtb enzyme. Of note, the Asp to Asn variant occurs naturally in the broad-spectrum amino-acid racemases (Bsrs), which in our analysis also show a negative rotamer for Asn.^17^ This observation suggests that the D322N mutation may also affect substrate specificity in MtAlr and other Alrs. However, no data are currently available, nor it is known whether Bsrs are less susceptible to DCS than Alrs.

In agreement with the UV–vis and fluorescence spectra, the structure of DCS-treated MtAlr D322N shows that the external aldimine **3** is the dominant species present at equilibrium. On the basis of the fluorescence data, we can estimate that less than 10% of active sites contains isoxazole **5**. Since the revised Alr inhibition/reactivation mechanism (Scheme 1) indicates that aldimine **3** is the chemical species from which enzyme reactivation can occur,^9^ Alr reactivation should occur more frequently in the D322N variant than in the wild-type enzyme. During *in vitro* inactivation assays in the presence of an excess of DCS,^5^ the external aldimine formed is sufficient to inhibit the enzyme in the short term. In the bacterial cellular milieu, however, increased MtAlr D322N reactivation results in the observed DCS resistance phenotype.

## Supporting Information Available

Experimental Methods; X-ray data collection and refinement statistic (Table S1); Available PDB coordinates for Alr and DadX enzymes (Table S2); Salt bridges and rotamer values of Alr orthologs (Table S3); Fluorescence emission spectra at 520 nm (Figure S1); Fo-Fc electron density map for the ligands in the active site of DCS-inactivated D322N (Figure S2); Sequence alignment of Alr and DadX enzymes (Figure S3) (PDF)
