# Supplementary material for: Structure of the d-Cycloserine-Resistant Variant D322N of Alanine Racemase from Mycobacterium tuberculosis
Source: ACS Bio Med Chem Au. 2023 Mar 27;3(3):233–9. doi: 10.1021/acsbiomedchemau.2c00074 (PMC10288493; doi:10.1021/acsbiomedchemau.2c00074)

SUPPORTING INFORMATION FOR

Structure of the D-cycloserine-resistant variant D322N of alanine racemase from *Mycobacterium tuberculosis*

Cesira de Chiara[1]*, Gareth A. Prosser[1]†, Roksana Ogrodowicz[2] and Luiz P. S. de Carvalho[1,3]*

[1]Mycobacterial Metabolism and Antibiotic Research Laboratory, The Francis Crick Institute, London, NW1 1AT, United Kingdom. [2]Structural Biology Science Technology Platform, The Francis Crick Institute, London, NW1 1AT, United Kingdom. [3]Department of Chemistry, The Herbert Wertheim UF Scripps Institute for Biomedical Innovation & Technology, Jupiter, FL, 33458, United States.

†Present Address: Research Center Borstel, Leibniz Lung Center, Parkallee 1-40, Borstel, 23845, Germany

EXPERIMENTAL METHODS

Chemicals

Unless otherwise stated all the chemicals and the reagents were purchased from Sigma-Aldrich

Protein expression

Recombinant expression and purification of *M. tuberculosis* Alr mutant D322N was performed as described in Evangelopoulos *et al.*[1]

Fluorescence and UV/Visible spectroscopy

Fluorescence spectra were acquired using a Shimadzu Fluorometer RF5301 and a 0.5 x 1 cm path-length cell for absorbed and emitted light. The temperature was kept at 37 °C using a circulating water bath. Slit widths were 1.5/3 nm for intrinsic tryptophan fluorescence (λmax excitation 284 nm/emission 327 nm),1.5/10 nm for isoxazole (λmax 318 nm/357 nm), 1.5/ 20 nm for oxime (λmax 365 nm/454 nm), and 5/15 nm for aldimine (λmax 434 nm/520 nm). Protein concentration was in the range 2.5-5 µM in 50 mM sodium phosphate pH 7.5, 100 mM KCl. The occurrence of internal quenching under these conditions has been preliminarily checked and excluded. Spectra were corrected for buffer contribution.

UV/Visible absorbance spectra were acquired at 37 °C using a Shimadzu UV-2550 spectrophotometer (Milton Keynes, UK) equipped with a thermostatic cell holder and using a 1 cm path-length cell.

DCS Inhibition, Crystallisation and X-ray data collection

D322N was preliminarily inactivated by DCS over 24 h at 30 °C (D322N:DCS ratio 1:100) and then kept at 20 °C until drops were set up a few hours later.

Crystals of D322N in its uninhibited and DCS-inhibited form were grown at 20 °C from sitting drops of 0.2 µL formed by 0.1 µL of either uninhibited or inhibited mutant stock at 10 and 20 mg/mL, respectively, (in 20 mM Tris pH 7.0) and 0.1 µL crystallisation solution containing: 100 mM sodium MES buffer pH 6.2, 150 mM CaCl, 9% (v/v) PEG Smear Broad (Molecular Dimensions), 2 %v/v Glycerol for uninhibited, and 100 mM sodium MES buffer pH 6.2, 150 mM CaCl, 10.5% (v/v) PEG Smear Broad (Molecular Dimensions), 4.3 %v/v Glycerol for the DCS-inactivated D322N. Cryoprotection was provided by preparing a 4:1 mixture of the crystallisation condition with 100% Glycerol. The crystals were transferred into this cryoprotectant prior to freezing in liquid nitrogen for data collection. Diffraction data were collected at beamline I04 at the Diamond Light Source, U.K. Integration and scaling were performed by the automatic data-processing pipeline at Diamond using XDS (3dii mode), within the XIA2 expert system for X-ray diffraction data processing[2]. Crystals of both the uninhibited and the inactivated form belong to the tetragonal space group $P4_12_12$ with two molecules in the crystallographic asymmetric unit.

Structure determination and refinement

Molecular replacement was carried out with PHASER[3], using the coordinates of DCS-inhibited *M. tuberculosis* alanine racemase (PDB code 6SCZ) with ligands and solvent molecules removed. Models were improved and refined using COOT[4] and PHENIX[5]. A summary of the data collection and refinement statistics is given in **Table S1**. The loop including the catalytic Y273 in chain B of uninhibited D322N is disordered from I265 to D282 and could not be modelled due to missing density. Positive electron density was present for this loop in the post-molecular replacement map of DCS-inactivated D322N. Although this density was less well-defined than for site B, fitting of all the residues in site A was possible, however resulting in significantly higher B-factor values. The structure for this loop in site A closely resembles the one of the better defined corresponding loop in site B. The density for the adduct formed by PLP and the antibiotic is well-defined in both active sites (**Figure S2**). Figures were drawn using PyMol (The PyMol Molecular Graphics System, Version 2.0 Schrödinger, LLC).

REFERENCES

(1) Evangelopoulos, D.; Prosser, G. A.; Rodgers, A.; Dagg, B. M.; Khatri, B.; Ho, M. M.; Gutierrez, M. G.; Cortes, T.; de Carvalho, L. P. S. Antibiotic resistance evasion is explained by rare mutation frequency and not by lack of compensatory mechanisms. *bioRxiv* **2018**, 374215. DOI: 10.1101/374215.
(2) Winter, G. xia2: an expert system for macromolecular crystallography data reduction. *Journal of Applied Crystallography* **2010**, *43* (1), 186-190. DOI: doi:10.1107/S0021889809045701.
(3) McCoy, A. J.; Grosse-Kunstleve, R. W.; Adams, P. D.; Winn, M. D.; Storoni, L. C.; Read, R. J. Phaser crystallographic software. *Journal of Applied Crystallography* **2007**, *40* (4), 658-674. DOI: doi:10.1107/S0021889807021206.
(4) Emsley, P.; Cowtan, K. Coot: model-building tools for molecular graphics. *Acta crystallographica. Section D, Biological crystallography* **2004**, *60* (Pt 12 Pt 1), 2126-2132. DOI: 10.1107/S0907444904019158.
(5) Afonine, P. V.; Grosse-Kunstleve, R. W.; Echols, N.; Headd, J. J.; Moriarty, N. W.; Mustyakimov, M.; Terwilliger, T. C.; Urzhumtsev, A.; Zwart, P. H.; Adams, P. D. Towards automated crystallographic structure refinement with phenix.refine. *Acta Crystallographica Section D* **2012**, *68* (4), 352-367. DOI: doi:10.1107/S0907444912001308.

Table S1 |Data collection and refinement statistics (molecular replacement)

	MtAlr D322N (8AHW)	MtAlr D322N+DCS (8B8H)
**Data collection**	I04	I04
Wavelength (Å)	0.9795	0.9159
Space group	$P4_12_12$	$P4_12_12$
Cell dimensions		
a, *b*, *c* (Å)	165.32 165.32 57.76	163.94 163.94 57.11
α,β,γ (°)	90, 90, 90	90, 90, 90
Resolution (Å)	82.66 - 1.58 (1.58 - 1.61)*	57.96 - 1.78 (1.81 - 1.78)*
R_{merge}	0.048 (2.596)	0.152 (1.765)
$I / \sigma I$	21.5 (1.1)	10.2 (0.9)
CC1/2	1.0 (0.6)	0.998 (0.347)
Completeness (%)	100.00 (100.00)	100.00 (100.00)
Redundancy	26.6(25.6)	12.3 (10.4)
No. unique reflections	109555 (5439)	74924 (3664)
**Refinement**		
Resolution (Å)	47.35 - 1.58 (1.636 - 1.58)	51.84 - 1.78 (1.844 - 1.78)
No. reflections	109449 (10803)	74847 (7366)
R_{work} / R_{free}	0.2262/0.2342 (0.3566/0.3582)	0.1997/0.2232 (0.3980/0.4340)
No. atoms		
Protein	5241	5497
Ligand/ion	63	149
Water	281	343
B-factors		
Protein¬`	45.61	37.25
Ligand/ion	44.88	32.99
Water	41.45	36.35
R.m.s. deviations		
Bond lengths (Å)	0.008	0.015
Bond angles (°)	1.03	1.14
**Ramachandran plot (%)**		
Most favoured	97.7	96.9
Allowed	2.3	2.8
Outlier	0	0.3

*Values in parentheses are for highest-resolution shell.

Table S2 Available PDB coordinates for Alrs and DadXs structures. PDB codes in bold are for enzymes inactivated by DCS

Organism	Uninhibited	Inactivated and/or ligand-bound
**Gene: *Alr***		
M. tuberculosis	1XFC	**6SCZ**
C. glutamicum	2DY3	
G. stearothermophilus	1SFT	**1EPV**/1NIU, 1BD0/1FTX, 1L6F/1L6G, 2SFP , **1XQK**/1XQL(Y265F)
B. subtilis	6Q70	
S. lavendulae	1VFH	**1VFS**, 1VFT
S. coelicolor A3(2)	5FAC	**5FAJ,** 5FAG
E. coli	2RJG	**2RJH**
B. anthracis	3HA1, 2VD8	2VD9
E. faecalis	3E5P	**3E6E**
B. henselae	3KW3	
S. pneumoniae	3S46	
C. difficile 630	4LUS, 4LUY(K271T)	**4LUT**
A. baumannii	4QHR	
B. pseudofirmus OF4	5YYC	
P. aeruginosa		6A2F
**Gene: *DadX***		
DadX_*P. aeruginosa*	1RCQ	
DadX_*P. fluorescens*	2ODO	

Table S3 Salt bridges and rotamer values of wild-type and D322N MtAlr, SlAlr and GsAlr.

	Shortest two distances (Å) between any couple of 'N' and 'O' (**R375-D322**)	Shortest two distances (Å) between any couple of 'N' and 'O' (**R375-D294**)		χ1 of D322 or N322 (A/B)
Active WT MtAlr (1XFC) 1.9 Å	4.5/5.5 (A)<br>5.3/5.5 (B)	3.2/3.3 (A)<br>2.6 /2.7 (B)		+64.6<br>+60.3
Inactive WT MtAlr (6SCZ) 1.57 Å	2.9/3.7 (A)<br>2.9/3.7 (B)	4.8/5.8 (A)<br>4.8/5.7 (B)		+75.2<br>+73.6
Active SlAlr (1VFH) 2.0 Å*	4.7/7.7 (A)*	2.7/3.9 (A)*		+30.1
Inactive SlAlr (1VFS) 1.90 Å	3.1/3.2 (A)<br>3.0/3.1 (B)	2.9/2.9<br>2.9/3.0		-32.3<br>-30.1
Active GsAlr (1SFT) 1.90 Å	2.8/2.8 (A)<br>2.8/2.8 (B)	2.9/2.9 (A)<br>2.9/3.0 (B)		-32.9<br>-39.4
Inactive GsAlr (1EPV) 2.2 Å	2.8/2.9 (A)<br>2.6/2.8 (B)	2.9/3.0 (A)<br>3.0/3.1 (B)		-36.5<br>-30.1
	Distance between 'N' and 'O' (**R375-N322**) (Å)		Distance between 'N' and 'O' (**N322-D294**) (Å)	
Active Mt D322N (8AHW) 1.58 Å	4.0 (A)<br>4.0 (B)	4.5/5.0 (A)<br>4.7/5.2 (B)	2.8/3.2 (A)<br>3.1/3.4 (B)	-66.9<br>-66.2
Inactive Mt D322N(8B8H) 1.78 Å	4.3 (A)<br>4.0 (B)	4.8/5.3 (A)<br>4.8/5.2 B)	2.9/3.2 (A)<br>2.9/3.3 (B)	-69.7<br>-66.9

*Only one chain in the asymmetric unit.

Figure S1. Fluorescence emission spectra at 520 nm of wild-type and D322N MtAlr, by excitation at 434 nm. The emission is due to PLP bound to the catalytic lysine 44 via an internal aldimine linkage **2** (Scheme 1 of main text). The time course monitors the inactivation reaction initiated by binding of DCS and displacement of K44 from the aldimine linkage to PLP. The data have been fitted using an exponential decay function (f = y0+a*exp(-b*x)) providing a rate of 12.9 and 0.47 h^{-1} for wild-type and D322N variant.

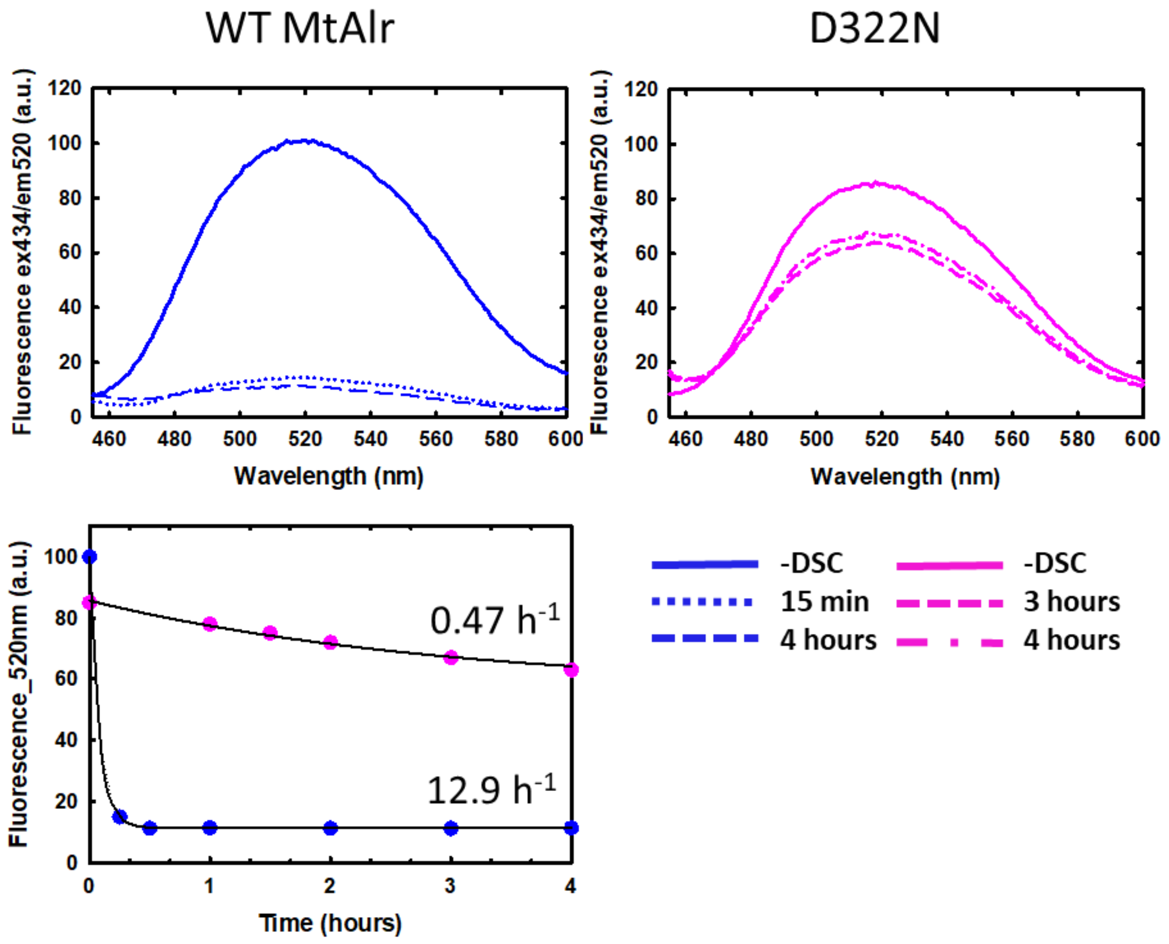

Figure S2. Positive electron density in the Fo-Fc map for the ligands in active site ‘A’ **(A)** and ‘B’ **(B)** of DCS-inactivated MtAlr D322N (PDB 8B8H) contoured at 2.5 σ. The omit map was computed at an early stage of refinement prior to the introduction of the ligand coordinates in the model. The superposed ligands from the final fully refined structure are shown as: **A)** a mixture of external aldimine **3** / isoxazole **5** with a large predominance of **3** in site ‘A’ and **B)** aldimine **3** as a sole species in site B. The bottom view is obtained by a 90° rotation along the shown axes.

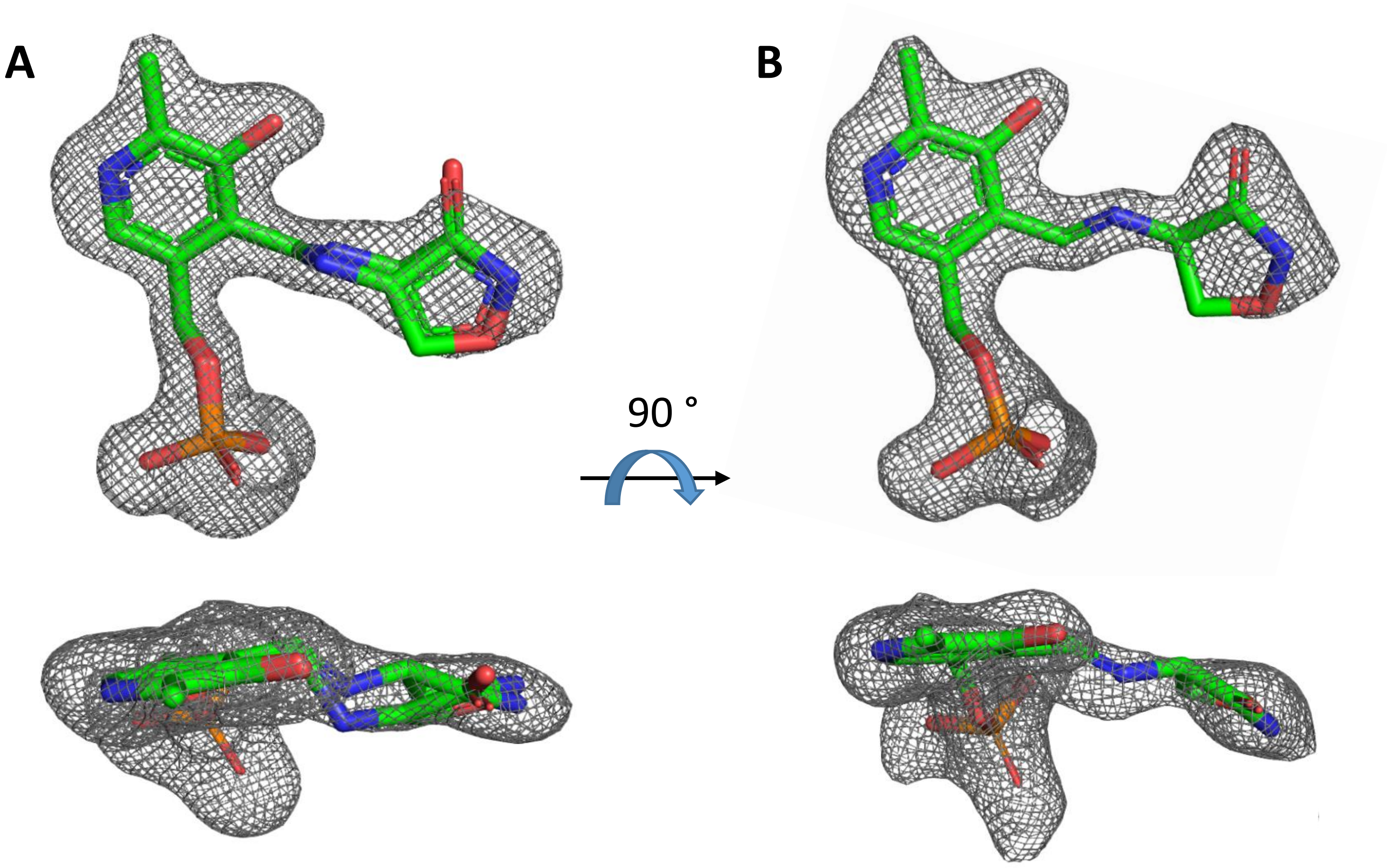

Figure S3. Sequence alignment of C-terminal stretches of anabolic alanine racemases Alrs and catabolic alanine racemases DadXs with structures available on the PDB as indicated in **Table S1**. Three additional sequences are shown for DadXs from *S. typhimurium* (UniprotKB P0A1A3), *E. coli* (UniprotKB P29012) and *P. putida* KT2440 (UniprotKB Q4ADX2**)**. Position of the conserved D294’, D322’, (in MtAlr) is highlighted by a red asterisk *, and R375’ by a black asterisk * (see also **Figure 3** of main text).

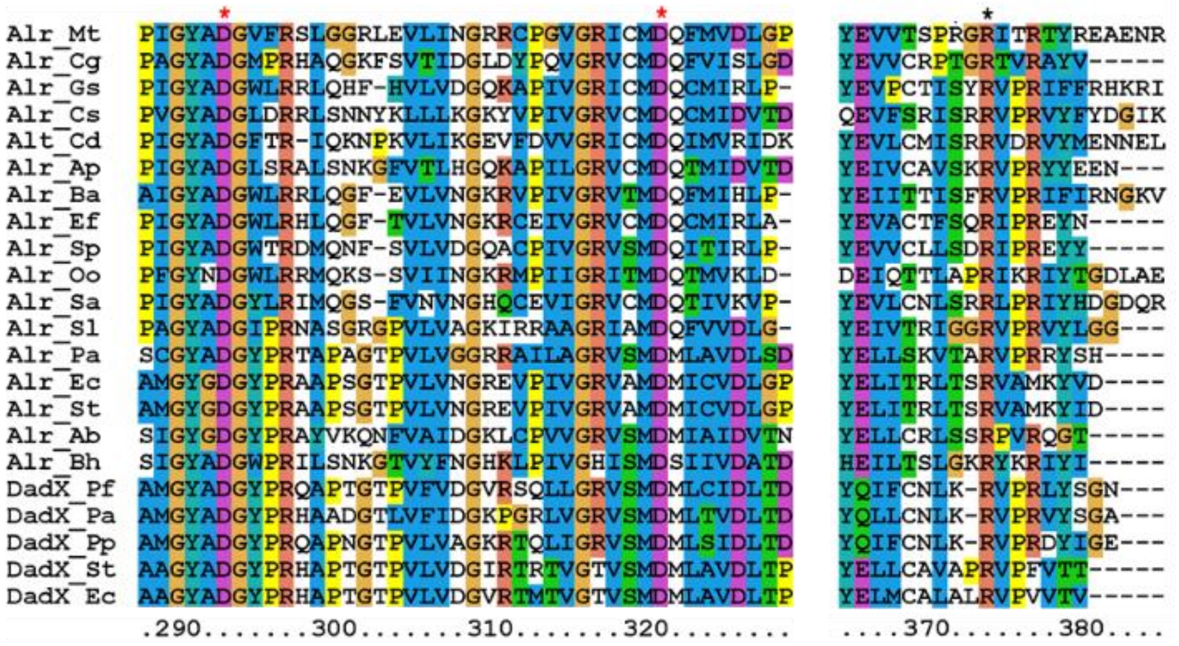

Supplement: Supplementary file 1 — bg2c00074_si_001.pdf [file bg2c00074_si_001.pdf]
